# Hospital and Institutional News

**Published:** 1916-05-27

**Authors:** 


### HOSPITAL AND INSTITUTIONAL NEWS.

### THE FAILURE OF THE RED CROSS CONFERENCE.

The Red Cross Conference which was to be held in Stockholm has completely broken down, and the principal object of the promoters has thereby been defeated. The ostensible cause of this regrettable event is the refusal of the German delegates to agree to a resolution expressing regret over the sinking of the Russian hospital ship *Portugal*. It will be recalled that the Germans—or the Turks on their behalf—issued a statement of that incident designed to create the impression that the submarine commander thought he was attacking a warship. Much that appeared in that statement was totally at variance with the official Russian accounts of what took place; and if it was really true, which no one believes, that the *Portugal* was torpedoed by oversight, we cannot imagine why the German Red Cross should not be willing to express regret for the error. However that may be, it is quite probable that the *Portugal* incident was not the main cause of the break-up of the conference. Part of the programme was the arranging of inspection committees for prison camps in the belligerent countries. The Hungarians and Austrians agreed readily to the proposals, but the Germans were bitterly hostile to the idea, and refused to entertain it, in spite of a visit to Berlin of a Swedish deputation in support. From what little is known already about Wittenberg and other places it is easily comprehensible that the publication of any further details about German treatment of prisoners, certified by neutral authorities, might not exactly suit the Huns.

### THE HENRY SAXON SNELL PRIZE.

The subject set for the medal of the Royal Sanitary Institute and the sum of fifty guineas, which constitute the Henry Saxon Snell Prize, was this year "Suggestions for the Improvement of Sanitary Arrangements and Sanitary Appliances on Shipboard"; and the successful essayist is Dr. William Hanna, Assistant Port Medical Officer for Liverpool. The subject set was a distinct stimulus to a section of the medical profession (medical officers of the Royal Navy and of the Mercantile Marine) which seldom gets a chance of academic distinction of this particular sort. Naturally, the experience of fifteen years in the Port Sanitary Service of so large a port as Liverpool was a valuable asset to Dr. Hanna in competing for the prize. We trust that his suggestions will be acted upon as far as possible both by those who design ships and those who order shipbuilding. There is ample room for improvement in sanitation on shipboard, and the Sanitary Institute was well advised in setting the subject for the essay competition.

### MEDICAL PRACTITIONERS AND THE MILITARY SERVICE BILL.

A very important new clause has been added to the Military Service Bill in the House of Lords. This clause enacts that there shall be established "professional committees to deal with claims for exemption made by duly qualified medical practitioners"; and that such claims brought before tribunals shall be referred to such a professional committee, whose decision shall be binding. We take it that the Central Medical War Committee and its local branches will be the recognised bodies thus contemplated, and that this clause represents the result of lobbying on behalf of that Committee, whose position will be immensely strengthened by this legislation. It does not appear that the clause is retrospective, which it might well have been made, for there are very strong rumours in the profession to the effect that a good many bachelor doctors of military age went before local tribunals and obtained total exemption when compulsion was applied to the single men a few months ago. If such exemptions were allowed to stand good while married men's claims were being disallowed by the Central Medical War Committee or its branches, there would certainly follow a great deal of excusable discontent in the profession. Indeed, there is urgent need for a reconsideration of the position of every medical man of military age who has not been abroad; for certainly there are cases of men who wear khaki but manage to put in only half-an-hour's work or so a day, and to look after their own practices for the rest of their time.

### SCHOLARSHIPS FOR WOMEN MEDICAL STUDENTS.

It is announced that Mr. S. Gamble has given £5,000 to Manchester University, to be devoted to assisting the education of needy women medical students. The scheme is to be administered by a small board of trustees, and the only conditions are that the women must have passed their first medical examination and be Christians and total abstainers.

### THE BRITISH HOSPITAL, PORT SAID.

Just before the war began, a decision was taken to rebuild on a much larger scale the old hospital which was first established at Port Said in 1886. The necessity for such a hospital, situated on one of the great trade routes, has grown ever greater with expansion of the carrying and passenger traffic to and from the East; and the outbreak of war has not relieved, but has increased, the urgency of the calls to which this institution responds. When the new scheme was adopted, the Egyptian Government gave £1,000 and a fine site; the Canal Company gave £1,000; and the shipping companies also subscribed liberally. In all, a sum of £16,600 has been collected for the rebuilding, and the structure is now in a sufficiently advanced state to receive patients, though much remains to be done to complete the equipment. The committee, of which the Hon. Arthur Stanley is chairman, is appealing for £4,400 still required to set the hospital going on an efficient basis, and an appeal is being issued to the British public for help towards this end. The influential committee of the hospital is sufficient guarantee that all funds entrusted to them will be well applied; and it gives us great pleasure to endorse this appeal, to which, we trust, a favourable reception will be given.

### THE PRINCESS LOUISE HOSPITAL.

Princess Louise, Duchess of Argyll, has contributed £1,000 towards the Scottish Hospital for Limbless Sailors and Soldiers, which is named after her. In forwarding the donation Her Royal Highness says it is delightful to see how kindly the Scottish public are taking up the movement, in aid of which some £70,000 has been subscribed in seven weeks. Further, it is announced that a Glasgow citizen, who wishes to remain anonymous, has acquired Erskine House and 350 acres of land, and has presented them to the hospital, which is evidently well on the way to a most successful start.

### THE LIGHTER SIDE OF LOOTING.

The Jervis Street Hospital is credited with having had to play the chief part in the demands made upon the hospitals during the recent disturbances in Dublin. Its central position gave it at once the place of danger which is the place of honour; and, according to a recent report, the hospital's staff received their first intimation of an outbreak from the arrival of two dead lancers at the institution. It is said that among those in need of assistance were numerous looters, and on this head it is interesting to add a report to the effect that looting at any rate was winked at by the authorities, who had their hands too full to make many arrests on this score. Out of the ruins of Sackville Street, however, arises more than one humorous tale, which we may advisedly nurse as an antidote to rancour, revenge, or all the other German futilities. For instance, people on the spot at the time have given extraordinary pictures of women who had never handled so much as an old gamp parading and posing amid the wreckage under a fashionable sunshade or in a "simply killing" hat. Black hands snatched at white gloves, and the make which did not split at this invagination would have a furore in Mayfair if its name could only be known. Even looting has its humours, and we need not be afraid of them once the mischief is over.

### THE PROPOSED SOUTH SHIELDS HOSPITAL IN SUNNYSIDE LANE.

Everything except a start has been given to the proposed new hospital for infectious disease, including tuberculosis, which the South Shields Corporation have had in mind for some time. Building may have to wait till after the war; but plans have been drafted by Mr. Leslie Roseveare, the borough engineer, in which he has attempted to embody the best features of the kindred institutions that he has visited. To be situated under the edge of Cleadon hills, on a slope which falls from southwards, the proposed hospital will accommodate 124 beds for cases of infectious disease and 66 beds for tuberculous patients. The former will have five pavilions, each of which, measuring 195 feet by 38 feet over all, will be fitted with verandahs roofed with glass. For the tuberculous patients a nursing pavilion measuring 338 feet long and three separate pavilions for men, women, and children will be provided. In addition, the children will have their own separate sheltered playground; while the men will have an opportunity for graduated exercise in a large parcel of ground set apart for cultivation. The buildings, including administration and laundry blocks, are to be built of brick, with facings of terra-cotta. From the reports published it is not clear if the nursing pavilion includes a special ward or wards for advanced cases. Such accommodation is necessary, but as a ward of this character is apt to get "a bad name" among the patients, its provision always raises some administrative difficulties.

### ELIZABETH FRY'S TRAINING SCHOOL.

In connection with the Florence Nightingale "Lamp Day," a correspondent of the *Morning Post* points out that the Institution of Nursing Sisters, founded in 1840 by Mrs. Fry, is still carrying on its work in a large Georgian house in an old-world square in the heart of the City. It was from this institution that Miss Nightingale picked out a party of nurses to accompany her to the Crimea. The institution is under the patronage of Queen Alexandra, and is in a flourishing condition: some at least of its work is done free of charge in cases of great necessity, thus carrying out the wishes and ideals of its foundress. The quaint rules as drawn up by Mrs. Fry are still read aloud with due solemnity to the sisters on their admission, and some of them are quoted in the letter to which reference has been made above. Once again, as sixty years ago, members of the institution are tending wounded soldiers both at home and abroad.

### A CURIOUS POINT IN SCOTTISH LAW.

After a good deal of litigation, the decision that a limited company may not style itself "Dr. Temple, Ltd.," has been upheld in Scotland, and the defenders eventually withdrew their defence and consented to a perpetual injunction. Curiously enough, however, the Lord Ordinary decided (and his decision has been upheld) that the prohibition applies to Edinburgh, but not to Glasgow. The reason for this apparently anomalous state of law, or of lawlessness, is that the Edinburgh College of Physicians brought the action against "Dr. Temple, Ltd.," but the corresponding authorities at Glasgow did not join in it. It would have been more satisfactory if the General Medical Council had been the complainants, for then the injunction would surely have applied to the whole kingdom: but the Scots law is so "pernickety" that possibly the General Medical Council preferred to let the Edinburgh College of Physicians undertake the necessary legal proceedings in defence of professional status and qualifications.

### PARISH COUNCILS AND TUBERCULOSIS WORK.

Difficulties have arisen in Scotland in connection with the care of certain classes of tuberculous patients, in consequence of the refusal of the Local Government Board to sanction further capital expenditure for the erection of sanatoria during the war, and because of the demand of the military authorities for all available hospital accommodation for wounded soldiers. At a conference of Glasgow and district local authorities under the Public Health Act, held recently, the Local Government Board suggested that where the resources of a local authority's hospital were exhausted, the parish council should receive the case into the poorhouse, and provide the ordinary maintenance, arrangements being come to whereby the local authority should be responsible for any necessary treatment. Although there is criticism on the ground of the overlapping likely to occur as a result of this dual responsibility, it is understood that the scheme is to be given a trial. Dr. Ebenezer Duncan, of Cathcart, is a strong opponent of the proposal, contending that the parish councils are not the authorities to have consigned to them the care of infectious cases.

### WALES'S NEW SANATORIUM.

The latest sanatorium to be opened in connection with the work of the Welsh Memorial Association is the new institution at Carnarvon, which has been officially inaugurated by Mrs. Lloyd George. Two years ago the institution was started in the town, but only in temporary quarters at the isolation hospital. It has now found a home at Bryn Seiont, formerly a private house, standing in two acres of ground under a mile from Carnarvon. There is accommodation for twenty-four patients and the necessary staff. In addition, however, detached wards for fifteen other patients have been established. These are made of timber frames with an external covering of corrugated sheeting, finished with pebble-dashed cement, and are lined inside with a patent form of plaster. The work has been carried out by Messrs. Humphreys, of Knightsbridge, whose temporary buildings are well known. The architect responsible for the work is Mr. Joseph Owen, of Anglesey. The Memorial Association, as Mr. D. W. Evans remarked at the opening ceremony, now possesses 481 "freehold" beds; it leases 180 more, and hires a further 200 from other institutions. Within three months the large sanatorium in the Vale of Clwyd is expected to be ready.

### A POINT FOR EDUCATION COMMITTEES.

An amusing observation is made in the annual report of Dr. W. H. Daley, the Bootle school medical officer, in a reference to the dental clinic and facilities for the operative treatment of adenoids and enlarged tonsils which have been added during the past year to the services of his department. Dr. Daley says it has been found necessary to shift the dentist's afternoon from Thursday to Friday because so many of his patients made holiday on the day after their teeth had been attended to. A Saturday solace does not, of course, depreciate the attendance records. Commenting upon the way in which the free meals are provided for necessitous children, the doctor suggests that an effort should be made to lay them properly, serve them nicely, and make them an education in how meals should be taken.

### THE DAY OF PEACE OFFERINGS

In most country cottages at the present time, and possibly in many town houses, a special plum-pudding is carefully stored, or rather hung on a peg in the larder, which will be boiled and eaten immediately on the declaration of peace. We must not join the fascinating game of prophecy as to the exact date on which it may be proper to consume these puddings; but we may remind hospital managers in advance that when that time arrives an opportunity will occur for getting over the difficulty created by postponed appeals. In peace celebrations as in war work the hospitals must take their due share, and one of the best ways for individuals to celebrate that great occasion will be by making a special peace offering to the hospital in their neighbourhood. Every hospital manager should think out in advance the best way of organising a peace offering collection in his locality.

### A FLY BIBLIOGRAPHY.

The literature of the house-fly has extended to such dimensions since responsibility was fixed upon the insect for carrying microbes which occasion various diseases, that it is interesting to have a medical officer of health's experience in investigating the subject thirteen years ago. Dr. J. T. C. Nash, medical officer of health for the county of Norfolk, in a recent address remarked how his attention had been drawn to the association of infantile mortality from diarrhœa with fly-infested houses so far back as 1901. After putting forward his views at a conference on infantile mortality at Chelmsford in 1902, he went to the British Museum to study the literature of the house-fly, and found that there was no British work extant on so common an object. The only publication which he had been able to find was a pamphlet by an American, dated 1872, and two Continental papers, bound together, dated 1796. Dr. Nash added that the first work on the biology of the house-fly was published by the University of Liverpool in 1907. Since then the stream of books has been flooding full and free. It includes, if we remember, an apology for the house-fly by a writer who felt that the anti-fly crusade was being overdone. The boom was a bore! The danger is a daily menace, and the crusade must not die with the vogue. Dr. Nash's remarks are welcome as putting before his hearers the new subject in proper perspective. The risk is as real as the chances of war, and may cost us in the long run far more casualties than the Germans. Now that summer has come, the fly has set in with its usual severity.

### THE ASYLUM WORKERS' ASSOCIATION.

This valuable organisation, which looks after the interests of the asylum attendants and nurses of Great Britain and Ireland, held its annual meeting on May 17. A large number of the members have joined the Colours, and it is not surprising that the Association, never opulent, became somewhat embarrassed financially. An appeal, however, to the vice-presidents and life members was so successful that not only was 1915 duly surmounted, but also the current year is faced without serious cause for anxiety. During the year Dr. James Nicoll, the editor of the Association's organ, the *Asylum News*, resigned his office; and the energetic and popular honorary secretary, Dr. J. F. Powell, is editing the paper with the assistance of Drs. R. Brown and Park Inglis. The usual monthly publication has been given up in favour of a quarterly issue, owing to war difficulties. The work of this Association is deserving of every support from the asylum workers whom it exists to serve: the subscription is only one-and-six a year, and it should command a larger membership amongst them than it appears to possess.

### THE EDITORIALS IN WAR HOSPITAL MAGAZINES.

It is much harder to write a good preface than to write a good book, and the experienced reader knows from his observation of this fact that a glance at the preface is enough to show the quality of any book which has one. It is the same with "editorials," of which a fine crop has come our way since the war hospital gazettes became popular. A good editorial promises a good magazine, for it means that, like the good preface, it has been written after, and not before, the drafting of the first issue's "make-up." This is the secret of a good editorial. There lies before us, as we write, the first number, and consequently the first editorial, of the *Gazette* of the 4th Southern General Hospital, Plymouth. Wanting to write history, as well as to make it (proceeding, that is, from the commoner to the rarer task), the *Gazette* of the 4th Southern General Hospital aptly begins with an account of its own birth, and an historical sketch of the manner in which the hospital arose to supplement the local Volunteer movement. Its first camp, we learn, dates as far back as 1902, so presumably it is one of the senior mushrooms which grew so vigorously and so well to meet *Der Tag* when it came. The next date given is August 4, 1914; and we think the writer, "Gaius," under-estimates the interest of history in deliberately omitting the connecting links between. All sins of commission have been forgiven to the historian, but the sin of omission must not be his; for if he does not remember, forgetfulness or invention will step into his niche and topple out his statue.

### THE LIGHTER SIDE OF THE WAR HOSPITAL.

A good letter, signed E. W. Evans, C.F., reminds us all that doctors need a lighter side in these war hospital magazines quite as much as patients. One way to supply this is to coax the patients and the staff to send to the office the innumerable caricatures (in prose, verse, and line) with which each class privately beguiles the tedium of convalescence or off-moments. An editor's rule should be that nothing is likely to be worth publishing which somebody would not call "unfit for publication." Standing between the gay caricaturist and the stern upholders of the strictest propriety, and armed withal with a blue pencil, any editor worth his salt can play off these two very successfully against each other. The art of doing this in such a way that the chance of friction is dissolved in the certainty of laughter is the art of editing. The art of sub-editing consists in dressing the window. May we therefore venture to suggest that more boldly printed headlines would improve the second number? For headlines attract, while "copy" should retain, attention. We have read this magazine all through, and enjoyed every page of it. But the headlines did not assist our enjoyment for the simple reason that the headlines, editorially speaking, were not there. The moral is large letters for tired readers. They were easier to learn from in childhood and are easier to read later in life. They do not scare the eye but save it trouble, and are therefore useful to sick patients and a tired, because a busy, staff.

### INFANT AND MATERNAL WELFARE WORK IN LIVERPOOL.

The Medical Officer of Health of Liverpool, Dr. E. W. Hope, is a strong adherent to the doctrine of heredity. However much of the original Adam the parents pass on to their children, the physical and social welfare of the offspring are at the present moment of vital interest to the nation as a whole, and a properly conducted infant and maternal welfare scheme, comprising as it does the whole scale of municipal activities directed to the advancement of the public health, is entitled to every encouragement that can be afforded it by the press and the public. After the first and paramount claim upon the public purse for the purpose of winning the war has been met in no direction is money better expended than on child welfare. In Liverpool the mortality-rate of infants under one year of age has been decreasing in the various districts of the city, but although this fact gauges the progress of preventive measures, it strengthens no case for the relaxing or limitation of present effort. The Liverpool municipality, acting under a plan of carefully co-ordinated work in conjunction with voluntary associations, is making every effort to study the problems brought to light by statistics, and to secure that want of improvement will not be caused by any lack of skilled endeavour to secure it. The report under review claims that a persecuted race, to which through its troubles the economic value of infant life has been brought home, often survives its persecutor. Infant welfare and ante-natal hygiene go far to solve this "riddle of the ages."

### EXTRA HEALTH VISITING AT MANCHESTER.

Chiefly because a serious epidemic of measles has made more efficient inspection necessary, the Manchester Corporation, by a large majority, has decided to appoint four additional women health visitors. They are to receive salaries of £104 a year, with an allowance of £5 for uniform. When objection was taken to this expenditure at the City Council meeting last week, it was stated that with the continual movement of soldiers home on leave, and the merging of several families into one house, the committee could not afford to delay taking action.

### SCHOOL INSPECTION IN DEVON.

There must be some lack of energy, or of tact, to explain the unsatisfactory figures of refusals by parents to allow the treatment of defective children in Devon, disclosed at a recent meeting of the Devon Education Committee. In no fewer than 456 cases parents declined to allow dental treatment of their defective children: out of 1,587 children examined, 1,423 were found to have defective teeth—a dreadful proportion—and about a third of these must go from bad to worse because their ignorant and stupid parents will not let them be treated. At the same meeting a proposal to prosecute in twenty-two cases where parents declined to allow the (gratuitous) treatment of grave defects of eyesight in their offspring was opposed on the ground that the 456 dental defaulters ought logically to be prosecuted also. Eventually the proposal was carried, on the understanding that further efforts at persuasion would first be employed: possibly when backed up by the hint of prosecution persuasion may prove more powerful in moving these intractable conscientious-objectors-to-their-children's-health from their obstinacy.

### THE FRUITS OF RESEARCH IN THE DUSTBIN.

By an ingenious analysis of house refuse Dr. J. T. Neech, the medical officer of health for Halifax, has submitted a scheme for the recovery of the useful ingredients in it which he lately submitted in a report to his committee. The analysis, which was the work of Mr. Hamilton, the Leeds city commerical manager, disclosed that 43 per cent. of house-refuse is dust, 33 per cent. is composed of cinders, 13 per cent. of paper and cardboard, and the rest of old tins, bottles, or unclassified rubbish. If this proportion be general, as it probably is, in large industrial towns, Dr. Neech argues that if the 6,000 tons of unburnt cinders could be separated from the 18,000 tons of house-refuse which are deposited every year in the Halifax Corporation tip at Birks Hall, they "would be worth half the price fetched for coke." At the current price of coke (18s. a ton) this would equal a saving of nearly £3,000, and he considers the process could be accomplished without much difficulty if the refuse could be passed over "a mechanically operated screen of sufficient length and with at least three meshes." Two or three sorters, it is suggested, could be employed in picking out anything of value while the refuse was passing over the screen. He puts the cost of the plant at a few hundred pounds, though a depôt would soon be necessary. The cinders could be sold, the paper or cardboard either burnt or torn into fine pieces and mechanically mixed with cinders and pressed into briquettes, which should find a ready market. The scheme appears to be at once scientific, economical, and productive. It is a capital instance of the value of what is certainly that side of scientific research which is least open to criticism—namely, research into the elimination of waste products and into the best means of converting these into a useful purpose. The municipal dustbin does not sound so imposing as the Research Laboratory. But when we come to results it more than holds its own.

### PANEL CHEMISTS AND THEIR REMUNERATION.

An important conference of pharmacists, attended by delegates from all parts of England and Wales, was held in London last week, under the chairmanship of the President of the Pharmaceutical Society, mainly for the purpose of considering what steps it was advisable to take in order to secure more equitable terms for the supply of medicines under the Insurance Act. It was resolved that negotiations be entered into with the Insurance Commissioners, with the object of increasing the remuneration either by an increase in dispensing fees or establishment charges or both, and that the decision of the Commissioners should be submitted to the local pharmaceutical committees in time for a ballot of panel chemists to be taken on the question as to whether or not they are prepared to continue on the panel for 1917. There can be no doubt that panel chemists have a real grievance in this matter; but they have themselves to blame for accepting terms which their colleagues in Scotland refused to accept. It is extremely doubtful, however, whether the panel chemists of England and Wales would be prepared to adopt the policy which secured the Scottish pharmacists payment on a more generous scale—namely, that of refusing to undertake insurance dispensing unless it were made reasonably profitable. It is even doubtful whether in the event of a ballot in favour of a "strike" it would be possible to secure such a course of action throughout the country.

### VAGRANCY IN SURREY.

The difficulty of inducing Guardians voluntarily to enter into schemes for the co-ordination of their work over large areas was well illustrated at a recent meeting of the Kingston-on-Thames Board of Guardians. The Surrey Vagrancy Committee have drawn up a scheme for the uniform administration of the casual wards in the county, providing for their transference from the Guardians to the committee. The committee points out that since the transfer of the casual wards in the Metropolis to the Metropolitan Asylums Board in April, 1912, seventeen out of twenty-four of them have been closed. On the other hand, in Surrey the decrease in vagrancy between 1911 and 1914 amounted to only 6 per cent. Since the war there has been a very general and marked decrease in the number of vagrants throughout the whole of England. The experience in London suggests that uniform control of casual wards over a large area has a marked effect in diminishing vagrancy. After the war vagrancy is likely to increase, and the question of its control by uniform administration will again become of great importance. The committee, therefore, asked the Kingston Guardians to join in the proposed scheme for Surrey. The Guardians decided not to do so by a very large majority, on the grounds that their powers should not be diminished. An important reform is, therefore, likely to be frustrated because a Board of Guardians considers its dignity would be diminished by it. The remedy lies with the Local Government Board, which has compulsory powers in the matter.

### THE INSPECTION OF MASSAGE ESTABLISHMENTS AND LYING-IN HOMES.

The London County Council have not lost much time in using the new powers recently conferred upon them in respect to the inspection of massage establishments and lying-in homes. The Public Control Committee of that body report that they have granted 567 applications for the registration of persons or companies carrying on establishments for massage or special treatment. Orders refusing registration have been served in fifteen cases, but appeals against three of the orders are pending. In the case of lying-in homes, 118 persons have been registered. In nineteen cases notices were served requiring alterations or repairs to premises, and in four cases the premises have been registered after this order was complied with.

### THE COURAGE OF DUBLIN HOSPITAL STAFFS.

The Board of Governors of the Richmond, Whitworth, and Hardwick Hospitals, Dublin, has passed unanimously the following resolution:—" The Board of Governors, at their first meeting after the recent rebellious outbreak, wish to place on record their high appreciation of the devotion to duty displayed by the members of the senior visiting staff—Doctors O'Carroll, Sir T. Myles, and Boyd. During the entire period of disturbance they remained night and day at the hospital doing everything that was possible for the injured, and by their example and guidance safeguarding the hospital and showing in the face of extreme danger and difficulty a loyal attachment to their duty, which the Board feel will ever live as an honour to them personally and to their profession." This resolution was passed after it had been decided to place on the minutes a letter received from three members of the visiting staff recording the courage of every section of the staff during the crisis.

### INCREASED PATIENTS IN INFIRMARIES.

The annual statement of Dr. Baly, medical superintendent of the Lambeth Infirmary, denotes the great additional work that has been thrown on Poor-Law infirmaries owing to the war. In the year 1913-14 the number of patients was 4,141; in 1914-15, 4,473; and in 1915-16, 5,349, or an increase over the first figures of 1,208, or not quite 30 per cent. This very large increase entails a considerable amount of extra medical and clerical work. For example, over 4,200 certificates have been issued under the National Insurance Act, as compared with 1,850 in the previous year. Dr. Baly states that it will probably be impossible to increase the medical staff, but further clerical assistance may become necessary, as the work was likely to become still greater. The records of the *x*-ray department show that 465 patients have been examined, including 286 in which photographs have been taken. The operations numbered 561, compared with 338 and 307 in the two preceding years, the increase being mostly made up of major operations. In the pathological laboratory 2,190 specimens have been examined. It is truly a remarkable record of the work done in a Poor-Law infirmary in war-time, when there are so many additional difficulties to contend with.

### FLAT RATES IN POOR-LAW INFIRMARIES.

Efforts are being made to induce the City of London and the Southwark Boards of Guardians to agree to pay a flat rate of a certain sum per day for the persons displaced from their institutions through the latter being taken over by the military authorities for wounded and sick soldiers. In both cases the Guardians have refused to depart from the original agreement to pay a certain amount per day and any extra sum required to cover the patients' cost at the end of each quarter when the cost is ascertained. Originally the Local Government Board laid down the principle that no Board of Guardians should make any profit out of the patients from another authority which had been forced to give up its institutions for the benefit of the country. A flat rate may produce a profit, however, as is shown in the balance-sheet of one Metropolitan Board of Guardians, which, at the suggestion of the L.G.B., took in boarders under this system of payment, with a resulting profit of over £600. It would be more equitable if the present arrangement were allowed to remain, though it does entail a little extra clerical labour—this being the reason—or rather one of the reasons—advanced for the suggested change.

### HOSPITAL REPRESENTATIVE FOR THE PHARMACEUTICAL COUNCIL.

The result of the Pharmaceutical Council election, which was declared on Wednesday, 17th inst., is of special interest to institutional pharmacists in that Mr. H. Skinner, chief pharmacist at the Great Northern Central Hospital, has been elected a member of the Council. This is the first time in the history of the Pharmaceutical Society that a pharmacist actually engaged in institutional work has been elected a member of the Council. It is, of course, well known that the President, Mr. E. H. White, was formerly chief pharmacist at St. Thomas's; his election to the Council was, however, not effected until after he resigned institutional work to pursue his career in the wholesale chemical trade. The many problems affecting pharmacists engaged in hospital work should receive more attention by the Pharmaceutical Council now that public pharmacy is directly represented.

